# Mechanical Enhancement and Fracture Mechanisms of SLA Photopolymer Composites Reinforced with Fish Bone Ash

**DOI:** 10.3390/polym18111348

**Published:** 2026-05-29

**Authors:** Cem Alparslan, Mert Minaz, Erhan Baysal, Muhammed Fatih Yentimur, Oğuz Koçar, Şenol Bayraktar

**Affiliations:** 1 Department of Mechanical Engineering, Faculty of Engineering and Architecture, Recep Tayyip Erdoğan University, Rize 53100, Türkiye; senol.bayraktar@erdogan.edu.tr; 2 FDM and Metallography Laboratory, Department of Mechanical Engineering, Recep Tayyip Erdoğan University, Rize 53100, Türkiye; 3 Department of Aquaculture, Recep Tayyip Erdoğan University, Rize 53100, Türkiye; mert.minaz@erdogan.edu.tr; 4 Alapli Vocational School, Zonguldak Bülent Ecevit University, Zonguldak 67850, Türkiye; erhanbaysal@beun.edu.tr; 5 Department of Civil Engineering, Faculty of Engineering and Architecture, Recep Tayyip Erdogan University, Rize 53100, Türkiye; muhammedfatih.yentimur@erdogan.edu.tr; 6 Department of Mechanical Engineering, Faculty of Engineering, Zonguldak Bülent Ecevit University, Zonguldak 67100, Türkiye; oguz.kocar@beun.edu.tr

**Keywords:** stereolithography, bio-waste, sustainable filler, additive manufacturing, photopolymer composite, salmon fish bone ash

## Abstract

In this study, salmon fish bone waste from the fish processing industry was converted into an inorganic ash filler by calcination and incorporated into an SLA-compatible photopolymer resin at 4, 8, and 12 wt.%. To compensate for filler-induced optical scattering and rheological changes, the printing parameters were systematically optimized, and the optimum conditions were identified as a layer thickness of 30 µm and an exposure time of 12 s. Tensile tests performed in accordance with ASTM D638 Type IV showed that fish bone ash significantly enhanced the tensile strength of the photopolymer matrix, increasing it from 24.8 MPa for the neat resin to 37.95 MPa at 12 wt.% filler loading. In contrast, increasing filler content reduced elongation at break and promoted a more brittle fracture response. Statistical evaluation using Welch ANOVA and Games–Howell post hoc analysis confirmed that filler loading had a statistically significant effect on tensile strength (*p* < 0.05). FTIR analysis revealed that the filler remained chemically stable within the matrix and that the interfacial interactions were predominantly physical rather than covalent. SEM observations indicated that low and medium filler loadings improved crack deflection and energy dissipation, whereas particle agglomeration at higher loading increased the tendency for brittle fracture. These findings demonstrate that fish bone ash can be used as a sustainable bio-waste-derived reinforcement to improve the mechanical performance of SLA photopolymer composites.

## 1. Introduction

Additive manufacturing (AM) technologies have attracted significant attention in the fields of materials science and engineering in recent years due to their advantages, such as design freedom, the ability to produce complex geometries, and reduced material waste [1,2,3]. Among these technologies, stereolithography (SLA)-compatible photopolymer resin stands out due to its high surface quality, dimensional accuracy, and fine detail resolution [4,5]. The SLA method is based on the layer-by-layer solidification of liquid photopolymer resins through controlled exposure to light, and this property makes it widely used in the production of functional polymer parts [6,7]. However, the mechanical performance of parts produced with SLA has significant limitations, particularly in terms of tensile strength, ductility, and fracture behavior. Pure photopolymer resins generally exhibit brittle fracture, limiting their use in load-bearing applications. Therefore, research aimed at improving the mechanical properties of SLA-based photopolymer systems has increased in recent years [8,9]. In the literature, it is observed that composite structures are formed by adding fiber, ceramic, and mineral-based filler materials into photopolymer resins for this purpose. For example, on the ceramic side, it has been shown that the dispersibility and interfacial bonding of particles such as Al_2_O_3_ within the photopolymer matrix can be improved with silane-based surface modifications; this is decisively beneficial for both rheological stability and mechanical performance [10]. Regarding mineral/particulate fillers, it has been reported that silica-derived fillers (synthetic amorphous silica, ground glass fiber, etc.) can improve the hardness and flexural properties of commercial SLA resins; the effect is sensitive to the type of filler and the loading ratio [11]. Similarly, it has been reported that the addition of glass beads coated with acrylate-based photopolymer resin significantly affects mechanical outputs such as bending/compression, as well as printability and fracture behavior [12]. In fiber-reinforced approaches, fiber orientation and distribution are challenging in SLA processes; however, it has been shown that tensile behavior can be optimized in resins developed with fibers such as Kevlar, along with parameters such as layer thickness and printing direction [13]. Bio-based fillers and nano-reinforcements are also gaining prominence; for example, it has been reported that bio-derived additives such as lignin can have an improving effect on tensile strength and modulus of elasticity in SLA outputs even at low concentrations [14]. It is also noted that nanofillers such as graphene oxide can influence mechanical behavior at low weight ratios while remaining within a suitable viscosity range, but the risk of agglomeration increases at high ratios [15].

On the other hand, increasing environmental awareness and sustainable production approaches have made the transformation of biological waste into high-value-added materials a significant area of research [16,17,18]. The use of biological waste from agriculture [19,20], livestock [21,22], and the food [23,24] industry as filler or reinforcement elements in composite materials offers solutions to waste management problems and contributes to the development of environmentally friendly materials. In this context, fish bone (FB) waste, which is generated in large quantities in the fish processing industry, stands out as a bio-filler material due to its high calcium phosphate content and inorganic structure [25,26]. FB ash can be purified from its organic components through appropriate calcination processes, transforming it into homogeneous and fine particles; this ensures a more balanced distribution within polymer matrices [27,28]. Although there are studies in the literature showing that FB-derived filler materials improve the mechanical properties of conventional polymer composites, the use of FB ash in SLA-based photopolymer systems is still represented by a limited number of studies. In particular, comprehensive studies on the effects of reinforcement ratio on mechanical behavior and the microstructural evaluation of fracture mechanisms are insufficiently present in the literature.

In this study, composite structures were produced by incorporating salmon bone ash, a bio-waste-derived filler, into a stereolithography (SLA)-compatible photopolymer resin at different weight percentages (4, 8, and 12 wt.%). The mechanical performance of the produced samples was evaluated by tensile testing, while the fracture surface morphologies of all experimental groups were comparatively examined using scanning electron microscopy (SEM). Furthermore, Fourier transform infrared spectroscopy (FTIR) analyses were performed to investigate the functional groups and possible interfacial interactions between the salmon bone ash and the photopolymer matrix. Thus, the role of salmon bone ash in the photopolymer matrix, its effect on mechanical behavior, interfacial interactions, and fracture mechanisms were evaluated through an integrated approach. In addition, tensile strength data were statistically analyzed; since the homogeneity of variance assumption was not met, Welch ANOVA and Games–Howell post hoc tests were applied. In this respect, the study aims to demonstrate the potential usability of fish bone ash as a sustainable and functional reinforcement material in SLA-based additive manufacturing applications. Unlike conventional mineral or ceramic fillers such as silica, glass-derived particles, and alumina, salmon fish bone ash represents a calcium-phosphate-based filler obtained from fish-processing residues. The present study was designed as a laboratory-scale proof-of-concept investigation to evaluate its feasibility as a sustainable reinforcement for SLA-compatible photopolymer composites. Therefore, the study does not aim to assess industrial-scale filler availability or commercial production capacity, but rather to demonstrate the mechanical and microstructural potential of this waste-derived filler in SLA-printed composites.

## 2. Materials and Methods

### 2.1. Preparation of Salmon Bone Ash

**Figure 1:**
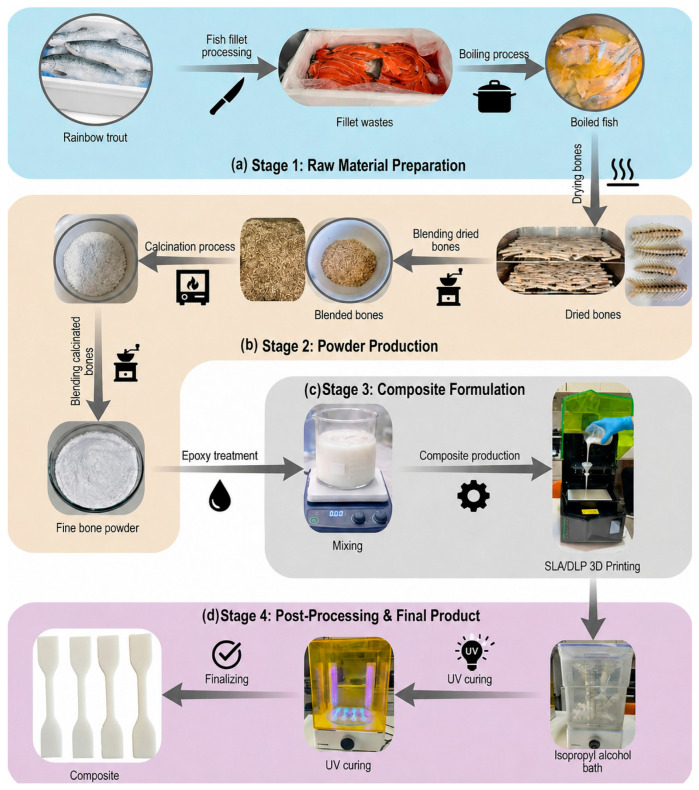
Workflow for producing bone-based composites from salmon waste: (**a**) raw material preparation, (**b**) powder production, (**c**) composite formulation and 3D printing, and (**d**) post-processing and final specimen fabrication.

Bone samples were obtained from fish fillets sourced from a specialized fish processing plant (Kuzuoğlu Group, Rize, Turkey). First, the fillets were boiled for approximately 30 min to remove the meat tissue from the bones. Following the boiling process, the bones were mechanically cleaned of any remaining soft tissue and washed with pure water. Then, the bone samples were dried overnight in an oven at 70 °C to remove moisture (Figure 1a). The dried bones were ground using a simple grinder for initial size reduction. Calcination was applied to completely remove organic components and obtain the inorganic phases of the bones (primarily calcium-phosphate-based components). Preliminary experiments were conducted to determine the calcination temperature, and the bones were subjected to heat treatment at different temperatures in 10 °C increments. As a result of these preliminary experiments, the ash samples obtained in the 600–900 °C range retained a dark or black appearance, suggesting the possible presence of residual carbonaceous matter or incomplete oxidation products rather than a fully purified inorganic ash. In contrast, calcination at 950 °C resulted in a white ash phase, indicating more complete removal of carbonaceous residues and the formation of a predominantly inorganic ash. Accordingly, the fish bones were held isothermally at 950 °C for 6 h. The result was a white salmon bone ash free of organic content. The main reason for choosing white ash obtained at 950 °C is that, when mixed with the photopolymer matrix under UV light used in the SLA production process, it provides sufficient light transmittance, allowing the resin to cure effectively. It has been observed that black ash obtained at lower temperatures increases light absorption, negatively affecting curing performance.

After calcination, the samples were cooled to room temperature and ground in a ball mill for 30 min to remove agglomeration and ensure homogeneous particle distribution (Figure 1b). The salmon bone ash prepared in this way was evaluated in subsequent stages for use as a reinforcement material in a photopolymer matrix. The product yield after calcination was not quantitatively determined, since the main focus was to evaluate the reinforcing effect of salmon bone ash in SLA-compatible photopolymer composites. Similarly, detailed quantitative chemical composition or phase analysis was not performed. However, based on the calcination process and FTIR findings, the obtained ash was evaluated as a predominantly inorganic calcium-phosphate-based filler. Future studies may include XRF, EDS, or XRD analyses to determine the elemental composition and crystalline phases of the ash in detail.

### 2.2. Preparation of Photopolymer/Bone Ash Composite Resins

A commercially available SLA-compatible UV-curable photopolymer resin (Anycubic Standard Resin) was used as the matrix material. This resin, developed for SLA/LCD-based additive fabrication systems operating in the 365–410 nm wavelength range, was chosen as a suitable matrix material in experimental composite studies due to its properties such as low viscosity, good fluidity, and dimensional stability. The resin was used as supplied by the manufacturer and no chemical pretreatment was applied [29]. Photopolymer/bone ash composite resins were prepared with salmon bone ash content of 4%, 8%, and 12% by weight as the reinforcing phase. The filler ratios were determined based on the ratio of the mass of the reinforcing phase to the total mass of the composite system. Accordingly, the filler ratio by weight (wf) for each composition was calculated using the following equation:

(1) $$w_{f}\left(\%\right)=\frac{m_{BK}}{m_{BK}+m_{R}}\times 100$$

Here, $m_{BK}$ represents the mass of salmon bone ash, and $m_{R}$ represents the mass of photopolymer resin.

The amounts of bone ash corresponding to the targeted filler ratios were calculated using the above equation, and the obtained values were weighed using an analytically precise balance. The weighed bone ash particles were added to the photopolymer resin matrix gradually and in a controlled manner. This approach was chosen to ensure homogeneous distribution of the filler phase within the resin, to prevent sudden agglomeration formation, and to maintain the rheological stability of the mixture. The mixture was stirred simultaneously during the filler addition process; this aimed to enhance the interaction between the reinforcement phase and the matrix, and to obtain reproducible and comparable composite resins at different filler ratios. The mixture was stirred simultaneously during the filler addition process to enhance the interaction between the reinforcement phase and the photopolymer matrix and to obtain reproducible composite resins at different filler ratios. The mixing process was carried out using a mechanical mixing device under ambient laboratory conditions while avoiding direct light exposure as much as possible to prevent premature resin curing. During mixing, salmon bone ash was added gradually in small portions to promote uniform wetting of the particles by the resin and to reduce the risk of sudden particle clustering. Mixing was continued until a macroscopically homogeneous suspension was obtained, as indicated by the absence of visible particle agglomerates or phase separation in the resin mixture.

After the mixing process was completed, the prepared composite resins were kept undisturbed for a short resting period before printing to allow entrapped air bubbles generated during mixing to rise and escape from the resin. This resting step was applied to reduce void formation and to minimize possible printing defects associated with trapped air. Prior to transferring the mixtures into the printing vat, the composite resins were visually inspected to confirm the absence of visible foam accumulation, large agglomerates, or macroscopic separation. Following this step, the composite resins were prepared for printing in the SLA-modified manufacturing process (Figure 1c).

### 2.3. SLA Printing Parameters and Specimen Fabrication

The samples to be used in mechanical tests were produced using an Elegoo Saturn 4 SLA-compatible resin-based printing system, which operates based on vat photopolymerization principles. Lychee Slicer 7.6.4 software was used to determine the pre-press slicing and production parameters. All printing processes were carried out in the same device and software environment to ensure that the variables in the process were kept under control.

Specimens to be used in tensile tests were designed in three dimensions using SolidWorks 2023 software, conforming to the Type IV geometry defined in the ASTM D638 standard [30]. The generated CAD models were exported in STL format and imported into Lychee Slicer software. The specimens were positioned in the software environment to be suitable for SLA production, and the support structures were automatically generated. A conical support geometry was preferred for the creation of the support structures; it was defined with a tip diameter of 0.6 mm, a tip length of 3.0 mm, and a body diameter of 1.3 mm. This support configuration was chosen to ensure geometric stability during printing and to minimize defects that may occur on the specimen surface.

Initially, trial prints were performed using the default resin printing parameters defined in the Lychee Slicer software for the Elegoo Saturn 4 device. However, it was observed that sufficient curing could not be achieved with these parameters due to optical and rheological changes in photopolymer/bone ash composite resins depending on the filler ratio. Therefore, a systematic optimization study was conducted on the layer thickness and exposure time parameters to improve print quality and interlayer bonding. In the parameter optimization process, layer thickness and exposure time were evaluated together using a linear approach. In this context, layer thicknesses were determined as 50, 45, 40, 35, and 30 µm, respectively, and the corresponding exposure times for each layer thickness were determined as 6, 8, 10, 12, and 14 s. Multiple trial prints were performed using the obtained combinations, and the print success was determined as follows: The samples were evaluated based on criteria such as complete curing of the layers, geometric integrity, surface continuity, and the absence of printing defects. As a result of this evaluation, suitable printing parameters for the production of photopolymer/bone ash composite resins were determined, and all mechanical test specimens were produced using these parameters. This approach resulted in the production of repeatable, comparable, and structurally integrated SLA specimens for composite resins with different filler ratios. The evaluations revealed that the most suitable printing parameters were a layer thickness of 30 µm and an exposure time of 12 s. This selection was based on the experimental observation that this parameter combination provided complete printing integrity, continuous specimen surfaces, and sufficient curing throughout the printed geometry. In contrast, the other tested combinations resulted in incomplete curing, reduced interlayer bonding, or visible surface defects, which made them less suitable for producing mechanically reliable test specimens.

After the printing process was completed, the resulting samples were cleaned using the Elegoo Mercury XS Bundle (Washing and Curing Station) system to remove any remaining uncured resin. The washing process was carried out in an isopropyl alcohol cleaning medium according to the manufacturer’s recommendations and was suitable for the chemical structure of the photopolymer resin. The aim of this stage was to completely remove any residual resin that might remain on the surface of the samples and to maintain surface integrity. Following the washing process, the samples were transferred to the curing unit of the same system and subjected to post-curing under UV light. The curing time was kept constant at 1 min, and the same conditions were applied to all samples. This process was carried out to ensure complete polymerization of the photopolymer matrix, increase mechanical stability, and obtain consistent material properties among the samples before testing. After the curing process, the samples were prepared for use in the mechanical and microstructural characterization stages.

### 2.4. Tensile Test Procedure

Tensile tests were performed on a WDW-5 brand universal tensile testing machine with a capacity of 5 kN to determine the mechanical behavior of the samples. The tests were conducted under static loading conditions and a constant head speed of 1 mm/min, and the ambient temperature was maintained at room temperature throughout the experiment. Basic mechanical parameters such as yield strength, tensile strength, and elongation to fracture were calculated through the applied load-deflection curves; the elastic modulus was determined from the linear portion of the elastic region.

**Figure 2:**
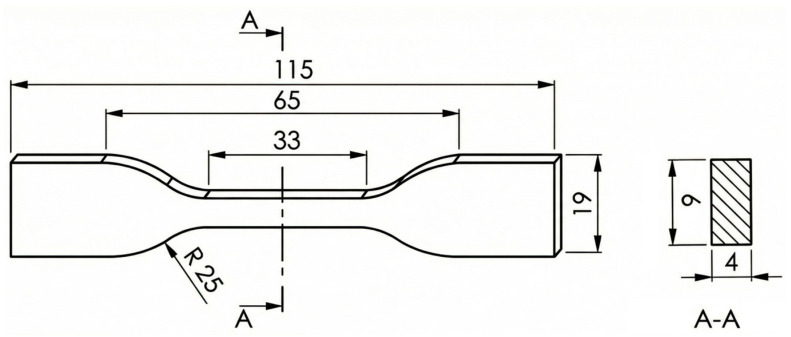
Shape and dimension of specimens for tensile test (Dimensions are in mm).

The tensile specimen was prepared according to ASTM D638-14 Type IV standard (Figure 2) [31,32]. Six replicates were performed for each experimental condition, and the data obtained were expressed as mean ± standard deviation to ensure the statistical reliability of the results. The repeated execution of the experiments allowed for the evaluation of inter-sample variations and improved the repeatability of the measurements. Throughout the test procedure, the axial alignment of the device and the jawing of the sample were meticulously performed; this aimed to minimize the effects of mechanical deviations such as shear, torsion, or off-axis loading on the measurements.

### 2.5. Fourier Transform Infrared Spectroscopy (FTIR)

Fourier transform infrared (FTIR) spectroscopy analyses were performed to determine the chemical interactions and functional group changes between the photopolymer resin matrix and fish ash. The analyses were carried out in ATR (attenuated total reflectance) mode using a Perkin Elmer Spectrum 100 FT-IR spectrometer (PerkinElmer, Waltham, MA, USA). Spectra were recorded in the wavelength range of 4000–400 cm^−1^ with a spectral resolution of 4 cm^−1^, and 32 scan averages were taken for each measurement. In the analysis, pure photopolymer resin, composite structures containing different weight ratios of photopolymer/FB ash (4, 8, and 12%), and pure photopolymer/FB ash were evaluated comparatively. Thus, interfacial interactions were interpreted through the observation of characteristic peaks of the filler phase in the matrix phase, changes in peak intensity, and possible peak shifts.

### 2.6. Fracture Surface Analysis of SLA-Printed Photopolymer Composite Parts

To investigate the interface morphology related to the fracture behavior of composite samples, fracture surfaces formed after tensile testing were analyzed using scanning electron microscopy (SEM). Imaging procedures were performed on an FEI Quanta FEG 250 field emission scanning electron microscope. Prior to imaging, the samples were sputtered with a thin layer of gold to reduce surface charging (which may arise from their dielectric characteristics) and to increase topographic contrast. Analyses were performed under high vacuum conditions and appropriate acceleration voltages; the distribution of the filler phase within the matrix, the level of interface bonding, particle pull-out, bridging behavior, and fracture modes were qualitatively evaluated from the microstructures obtained at different magnification levels. Thus, the morphological role of FB ash within the photopolymer matrix was revealed in a way that can be correlated with mechanical performance.

### 2.7. Statistical Analysis

In this study, experimental findings were evaluated using one-way analysis of variance (ANOVA) to examine the mean differences between single-factor groups. The ANOVA approach, pioneered by Fisher, is a common and standard statistical method for comparing group means in single-factor experimental designs involving three or more levels [33]. In this study, the photopolymer resin matrix used in SLA production and the tensile specimen geometry were kept constant, and only the effect of the weight percentage of salmon bone ash from biological waste (4, 8, 12%) on tensile strength was investigated. All statistical analyses were performed using IBM SPSS Statistics 29 software. Descriptive statistics (Mean, standard deviation, and 95% confidence interval) for tensile strength were calculated for each additive level; the normality assumption was tested with the Shapiro–Wilk test and Q–Q plots, and the homogeneity of variance assumption was tested with the Levene test. The valid application of one-way ANOVA depends on the assumptions of normality and homogeneity of variance; in particular, failure to ensure homogeneity of variance can weaken the reliability of the F-statistic [34,35].

Levene test findings showed that the variances were not homogeneous in terms of the tensile strength variable. Therefore, in this analysis, Welch one-way ANOVA, a more suitable and robust approach in cases of heterogeneous variance, was used, and the Welch F-statistic was used in interpreting the results [36,37,38]. When a statistically significant difference was found between groups (*p* < 0.05), the Games–Howell post hoc test, known to better control for Type I error rates under heterogeneous variance conditions and/or unequal sample sizes for pairwise group comparisons, was applied [39,40,41,42].

Throughout the study, the significance level was set at α = 0.05 for all hypothesis tests. The effect of salmon bone ash additive ratio on the tensile strength of SLA-based photopolymer composites was evaluated both statistically and from an engineering perspective by considering the F and *p* values obtained from Welch ANOVA, the corrected pairwise comparison *p* values obtained from the Games–Howell test, and 95% confidence intervals. With this approach, it was systematically determined whether the observed differences, while keeping the resin system and sample geometry constant, were due solely to the effects of the additive ratio, using multiple comparison procedures supported by assumption checks and suitable for heterogeneous variance conditions.

## 3. Results and Discussion

### 3.1. Processing Optimization of SLA-Printed Photopolymer/Bone Ash Composites

The addition of salmon bone ash to the photopolymer matrix caused significant changes in the optical and rheological properties of the resin, directly affecting the curing behavior in the SLA printing process. In the initial trial prints, conducted using the default printing parameters defined in the Lychee Slicer software for the Elegoo Saturn 4 device, it was observed that sufficient interlayer bonding could not be achieved in the composite resins, and partial curing problems occurred. This situation can be explained by the reduction in light penetration depth due to the scattering and absorption of UV light by the filler particles. It has been reported that the addition of fillers in photopolymer nanocomposites significantly reduces the UV penetration depth and directly affects printing success [43]. It is also known that filler particles, due to their high refractive index difference, increase light scattering, leading to heterogeneity in the curing profile. In particular, ceramic particles with a high refractive index have been reported to hinder UV light penetration, leading to irregular curing and reduced dimensional accuracy [44]. However, it has long been known that in photopolymer systems, the scattering phenomenon of the filler phase within the light beam can negatively affect print quality [45].

Therefore, a systematic optimization study was conducted on layer thickness and exposure time parameters to improve print quality and interlayer bonding. Layer thicknesses were determined as 50, 45, 40, 35, and 30 µm, and exposure times as 6, 8, 10, 12, and 14 s, respectively, and multiple test prints were performed. Print success was evaluated according to the criteria of complete curing of layers, geometric accuracy, surface continuity, and absence of print defects. Experimental observations showed that print quality improved significantly with decreasing layer thickness. It was determined that at thinner layer thicknesses, the surface quality improved and defects such as layer separation were eliminated due to the more effective spreading of light within the resin and more homogeneous curing of each layer. The literature emphasizes that in SLA processes, the curing depth is directly related to light energy and layer thickness, and that penetration depth is one of the fundamental parameters determining print success [46,47]. Increasing the exposure time supported the completion of the photopolymerization reaction, strengthening interlayer bonding. It is known that in systems where filler particles partially block light energy, a higher energy input is required, and curing quality is sensitive to exposure time [48].

The optimization study revealed that print quality continuously improved with decreasing layer thickness, and the most suitable surface quality and production stability were achieved at a layer thickness of 30 µm. Considering that curing behavior improved with increasing exposure time, a 12 s exposure time was chosen for the production of all composite samples. This parameter combination resulted in samples with high geometric accuracy, good surface continuity, and strong interlayer bonding.

### 3.2. Tensile Properties of SLA-Printed Photopolymer Composites

**Figure 3:**
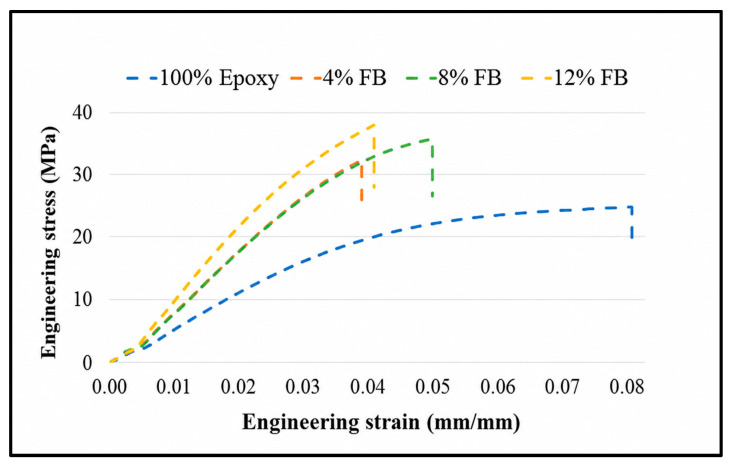
Representative stress–strain curves of the neat photopolymer and photopolymer/fish bone ash composite samples. The curves illustrate the general tensile behavior of each experimental group, while the quantitative mechanical values were calculated from six replicate specimens and are presented as mean ± standard deviation in Table 1.

The mechanical behavior of photopolymer-based composites produced by the SLA method shows significant variations depending on the presence and amount of the filler phase. In this context, the tensile behavior of pure photopolymer and composite samples containing different proportions of salmon bone ash was evaluated comparatively. The representative stress–strain curves shown in Figure 3 illustrate the general tensile behavior of each experimental group. All samples initially exhibited linear elastic behavior, followed by sudden fracture after limited plastic deformation. This behavior is consistent with the brittle fracture mechanism commonly reported in vat polymerization-based photopolymer materials [8,9].

The pure photopolymer sample exhibited the highest ductility with a maximum tensile strength of 24.8 MPa and an elongation of 8.37%. With the addition of FB, a significant increase in tensile strength was observed, while a general decrease in elongation values occurred. The improvement in tensile strength can be mainly attributed to the reinforcing effect of the rigid calcium-phosphate-based FB ash particles within the photopolymer matrix. These inorganic particles may act as load-bearing sites and contribute to the transfer of applied stress from the relatively brittle polymer matrix to the filler phase. In addition, possible physical interactions between the polar groups of the photopolymer and the phosphate/hydroxyl groups on the FB ash surface may improve matrix–filler compatibility, supporting more effective stress transfer during tensile loading. As a result, crack propagation becomes more tortuous, and part of the applied mechanical energy can be dissipated through crack deflection, particle–matrix interaction, and localized fracture path deviation. This mechanism is also consistent with the SEM observations, where the reinforced composites exhibited rougher fracture surfaces than the neat photopolymer. However, the simultaneous decrease in elongation indicates that the rigid filler particles restricted polymer chain mobility, thereby limiting deformation capacity and promoting a more brittle fracture response. Similarly, it has been widely reported in the literature that the addition of fillers to particle-reinforced photopolymer composites increases rigidity while decreasing ductility [14,15]. In composite samples containing 4% FB, the tensile strength increased to 32.4 MPa, showing an increase of approximately 30% compared to pure photopolymer. This increase can be attributed to the relatively homogeneous distribution of particles within the matrix and the efficient occurrence of the load transfer mechanism at low filler ratios. It is known that low filler ratios in particle-reinforced composites generally improve mechanical properties by strengthening matrix–filler interactions [10]. Increasing the filler ratio to 8% resulted in a tensile strength of 35.7 MPa and a 5.16% elongation, achieving the highest deformation capacity among the reinforced samples. This indicates that, at this composition ratio, the filler phase is more evenly distributed within the matrix, ensuring effective stress transfer. The literature generally reports that the optimum filler ratio is obtained in compositions where the particle distribution is homogeneous and interfacial bonding is maximized [12]. The highest tensile strength was obtained in the composite sample containing 12% FB, reaching 37.9 MPa. However, the elongation to fracture was 4.21%. This behavior observed at high filler ratios can be explained by increased particle–particle interaction and possible agglomeration formation, which may partially disrupt matrix continuity. Similarly, it is known that high filler ratios cause stress concentrations in composite systems, increasing the tendency toward brittleness [11,15].

When the results given in Table 1 are evaluated together, it is seen that the addition of FB significantly increases the tensile strength by strengthening the load transfer mechanism in the photopolymer matrix. However, increasing the filler ratio leads to a decrease in deformation capacity. This situation is consistent with the typical mechanical behavior of particle-reinforced composites. It has also been reported in previous studies that the matrix tends to embrittle, especially in biofilled systems with high mineral content [25,26]. Overall, the highest tensile strength was achieved at 12 wt.% FB, confirming the strengthening effect of the rigid inorganic filler phase. However, when tensile strength and deformation capacity were evaluated together, the 8 wt.% FB composition exhibited a more balanced mechanical response. Therefore, 8 wt.% FB should not be interpreted as the composition providing the maximum tensile strength, but rather as the optimum composition in terms of the strength–ductility balance. These results demonstrate that biological waste-derived filler materials can effectively improve the mechanical performance of SLA photopolymer systems, while the optimum filler ratio should be defined according to the targeted balance between strength, deformation capacity, and application requirements.

### 3.3. FTIR Analysis of Photopolymer/Bone Ash Composites

FTIR analyses, conducted to evaluate the chemical interactions and functional group exchanges between the photopolymer matrix and the FB ash, show that the filler phase remains chemically stable within the composite structure and forms interfacial interactions with the matrix. When the spectra of pure photopolymer, pure FB, and composite samples with different FB ratios (4, 8, and 12%) were examined together, it was determined that characteristic bands belonging to both the matrix and the filler phase were observed simultaneously in the composite systems.

**Figure 4:**
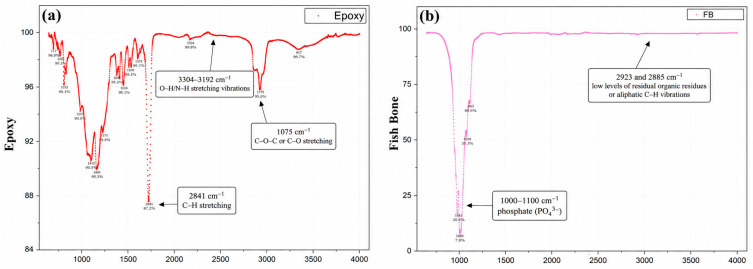
ATR-FTIR spectra of (**a**) photopolymer (epoxy) matrix and (**b**) fish bone ash, with the main characteristic bands assigned to O–H/N–H, C–H, C–O/C–O–C, and phosphate-related vibrations.

In the pure photopolymer spectrum, bands observed in the range of 3304–3192 cm^−1^ are associated with hydrogen bonding-sensitive O–H/N–H stretching vibrations, while the band observed around 2841 cm^−1^ corresponds to aliphatic C–H stretching vibrations. The 1075 cm^−1^ band, clearly observed in the fingerprint region, is consistent with C–O–C or C–O stretching vibrations in the photopolymer structure. These bands demonstrate that the chemical structure of the photopolymer matrix is preserved in composite systems (Figure 4a). The literature reports that in photopolymer resins, these bands represent the main chemical backbone of the matrix and are generally preserved with the addition of fillers, with only changes in band intensities observed [49]. The strong band observed in the spectrum of pure FB ash, approximately in the range of 1000–1100 cm^−1^, is associated with phosphate (PO_4_^3−^) vibrations belonging to the bioderived apatite structure (Figure 4b). This region is known as a characteristic spectral feature of hydroxyapatite and bone-derived bioceramics [50]. Furthermore, the bands observed around 2923 and 2885 cm^−1^ can be associated with possible low levels of residual organic residues or aliphatic C–H vibrations due to surface adsorption. These findings confirm that the inorganic character of FB is dominant.

**Figure 5:**
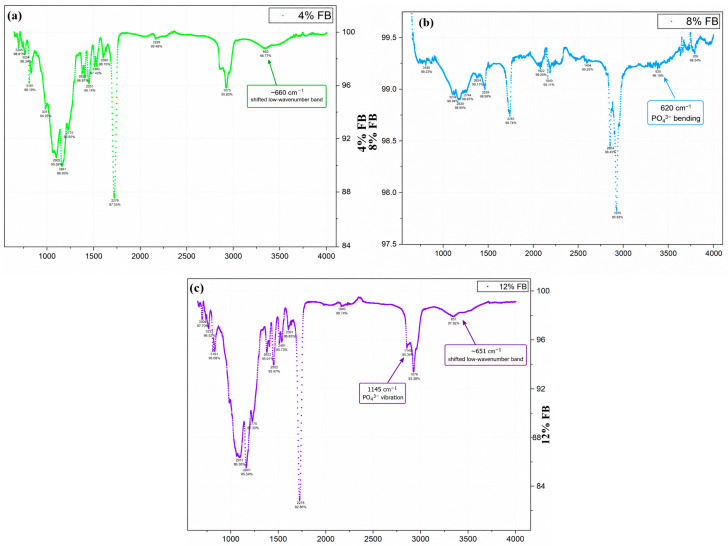
ATR-FTIR spectra of photopolymer/fish bone composites at different filler loadings, (**a**) 4 wt.%, (**b**) 8 wt.%, and (**c**) 12 wt.%, illustrating compositional effects on the fingerprint region and phosphate vibration intensity.

When the composite spectra are examined, it is observed that while the matrix bands are preserved, the bands belonging to the filler phase become more prominent as the filler ratio increases. In particular, the band observed around 1075–1076 cm^−1^ in all composite samples exhibits a mixed band character resulting from the overlap of the C–O vibrations of the matrix and the phosphate bands of FB. The prominence of the band observed around 1145 cm^−1^ in the 12% FB sample indicates that phosphate vibrations become more dominant with increasing filler ratio (Figure 5). Similarly, it has been reported in the literature that in polymer composites containing phosphate-based fillers, the intensity of the phosphate bands increases with increasing filler ratio, and the composite spectrum is shaped by these bands [51].

The shift in the band observed around 658 cm^−1^ in the photopolymer sample in the low wavenumber region to around 660 cm^−1^ and 651 cm^−1^ in the composite samples indicates a change in the local chemical environment between the matrix and the filler (Figure 5a). The 620 cm^−1^ band observed in the 8% FB sample is consistent with the phosphate bending vibrations of the hydroxyapatite structure (Figure 5b). These small band shifts are considered indicative of microenvironmental changes created by the filler particles within the matrix [52].

The preservation of hydrogen bonding-sensitive bands and the observed changes in bandwidths in the composite spectra indicate that weak interfacial interactions may occur between the hydroxyl and phosphate groups on the FB surface and the polar groups of the photopolymer matrix. Such physical interfacial interactions can improve matrix–filler compatibility and facilitate load transfer between the photopolymer matrix and the fish bone ash particles, thereby contributing to the improved mechanical performance of the composite system. However, the FTIR results indicate that these interactions are mainly physical in nature rather than being associated with the formation of new covalent bonds. Similarly, it is stated in the literature that matrix–filler interactions in polymer composites containing phosphate-based bioceramic fillers mostly occur in the form of hydrogen bonds and dipole interactions [53]. In conclusion, FTIR analyses show that FB ash remains chemically stable within the photopolymer matrix, and characteristic phosphate bands become more pronounced depending on the filler ratio. The preservation of matrix bands confirms that the photopolymer structure is not degraded during the composite production process, while band shifts and intensity variations indicate the presence of interfacial interactions. This supports the idea that the increase in strength observed in mechanical tests is related to the hard particle effect of the filler and the matrix–filler interface compatibility.

### 3.4. Fracture Surface Morphology

**Figure 6:**
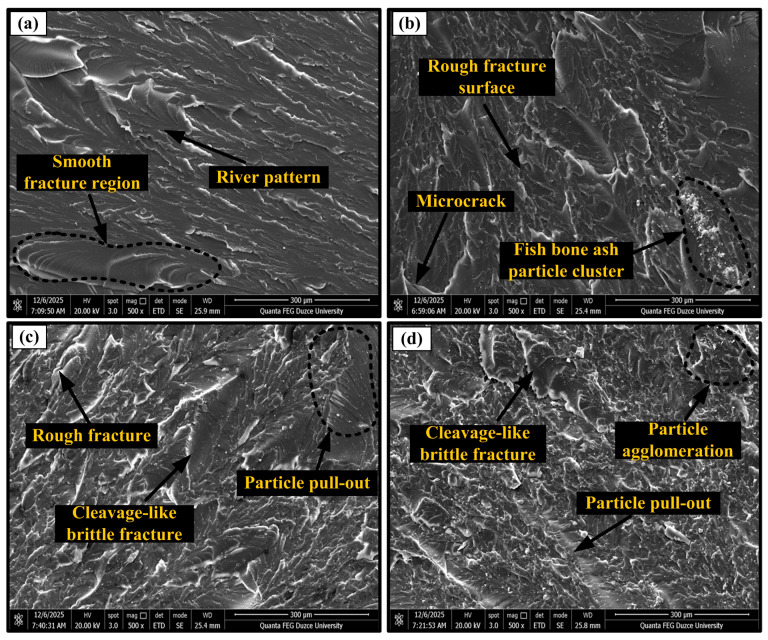
SEM images of fracture surfaces of photopolymer and photopolymer/fish bone ash composites with increasing filler content, (**a**) 100 wt.% photopolymer, (**b**) 4 wt.%, (**c**) 8 wt.%, and (**d**) 12 wt.%. Increasing filler loading leads to rougher fracture surfaces, particle clustering, pull-out features, and enhanced brittle fracture characteristics.

SEM analyses (Figure 6) clearly revealed the effects of different FB ash additive ratios in the photopolymer matrix on fracture morphology and damage mechanisms. The microstructural findings indicate that, depending on the filler ratio, the fracture behavior evolves from matrix-controlled brittle fracture to complex fracture mechanisms where particle–matrix interactions are prominent.

When the fracture surface of the pure photopolymer sample was examined (Figure 6a), a relatively flat and layered morphology was observed, indicating typical brittle fracture behavior. There are a limited number of plastic deformation traces on the surface, and it is understood that crack propagation occurred with low energy absorption. The literature reports that pure thermoset resins similarly exhibit low toughness and limited crack deflection [54,55]. In the composite sample containing 4% FB ash, the fracture surface was observed to become rougher and more irregular (Figure 6b). This morphological change indicates that the filler particles deflect crack propagation, thereby increasing energy absorption. The micro-protrusions and localized plastic deformation zones observed along the surface reveal that particle–matrix interaction contributes to load transfer. Many studies have reported that particle reinforcement, even at low rates, increases the fracture toughness of composites and hinders crack propagation [56,57].

Increasing the filler ratio to 8% resulted in a more complex morphology on the fracture surface and a significant increase in surface roughness (Figure 6c). In this sample, it is assessed that the particles are more homogeneously distributed within the matrix and that microcrack deflection and crack bridging mechanisms become effective. This indicates that the particles participate more effectively in load transfer and that the energy absorption capacity of the composite increases. The literature states that mechanical performance reaches its maximum with improved particle distribution at optimum filler ratios [58,59,60]. In the composite sample containing 12% filler, it was observed that the fracture surface became significantly heterogeneous, with particle agglomerations and microvoids forming (Figure 6d). This morphology indicates that matrix continuity is disrupted and interfacial bonding is weakened due to the high filler ratio. Agglomeration regions cause stress concentration, facilitating crack propagation and increasing the tendency for brittle fracture. It has been widely reported in the literature that increased particle–particle interactions at high filler ratios can lead to decreased mechanical performance [61,62].

Overall, SEM results show that the addition of FB ash significantly alters the fracture mechanism. At low and medium filler ratios, particle reinforcement exhibits a more robust behavior by supporting crack deflection and energy absorption mechanisms [63], while at high filler ratios, brittle fracture behavior becomes dominant due to particle agglomeration and weak interfacial zones [64]. These findings are consistent with studies showing that mechanical performance in particle-reinforced polymer composites is strongly dependent on filler ratio [65,66]. In conclusion, microstructural analyses show that FB ash reinforcement modifies the fracture behavior of the composites, and the optimum filler ratio is approximately in the intermediate range. This confirms that particle distribution and interfacial interaction are key parameters determining composite performance.

### 3.5. Correlation Between Mechanical Performance and Structural Features

Combined evaluation of mechanical results obtained from tensile tests and SEM analyses shows that the mechanical behavior of photopolymer/FB ash composites is controlled by the distribution of the filler phase, interfacial interaction, and filler ratio. Recent studies have also shown that the mechanical performance of particle-reinforced polymer composites is strongly dependent on the homogeneous distribution of the filler phase and the quality of interfacial bonding [67]. It has been observed that at a low filler ratio (4%), the particles are more homogeneously distributed within the matrix, and this can be attributed to an effective load transfer mechanism. It is reported that particle–crack interaction in nanocomposite systems improves the energy dissipation mechanism and increases strength by deflecting crack propagation.

The balanced mechanical behavior obtained at a medium filler ratio of 8 wt.% FB can be explained by the more regular particle distribution and sufficient interfacial bonding. Although the maximum tensile strength was obtained at 12 wt.% FB, the 8 wt.% FB composition provided a more favorable compromise between strength and deformation capacity. Therefore, in the present study, the term “optimum” refers specifically to the strength–ductility balance rather than the maximum tensile strength alone. This interpretation is consistent with the concept that, in particle-reinforced composites, the optimum filler ratio is often determined by the balance between mechanical strength, ductility, particle dispersion, and interfacial bonding quality [68]. Agglomeration and microvoid formation observed at a high filler ratio (12%) increased the tendency for brittle fracture by causing stress concentrations. It has been widely reported in the literature that increasing the filler volume ratio can increase rigidity while decreasing ductility, and that fracture behavior at high filler levels is controlled by particle aggregation [67,69].

FTIR results also support the existence of physical interactions between the matrix and the filler. It is stated that interfacial interactions control fracture behavior by affecting energy dissipation mechanisms in composites and are decisive in mechanical performance [70]. Overall, these study results are consistent with current literature showing that the mechanical performance of particle-reinforced polymer composites is strongly dependent not only on the amount of filler but also on the microstructural distribution and interfacial compatibility [67,68].

### 3.6. Statistical Analysis of Tensile Strength

The effect of FB ratio on tensile strength was evaluated using one-way analysis of variance (ANOVA) with material (100% Photopolymer, 4% FB, 8% FB, and 12% FB) as the independent variable and tensile strength (MPa) as the dependent variable. Descriptive statistics showed a significant upward trend in average tensile strength as FB content increased. The lowest average value was observed in the 100% Photopolymer group, and the highest average value was observed in the 12% FB group. However, the 12% FB group also showed the highest within-group variability; this indicates that the dispersion of experimental results increased at the highest FB ratio (Table 2).

**Figure 7:**
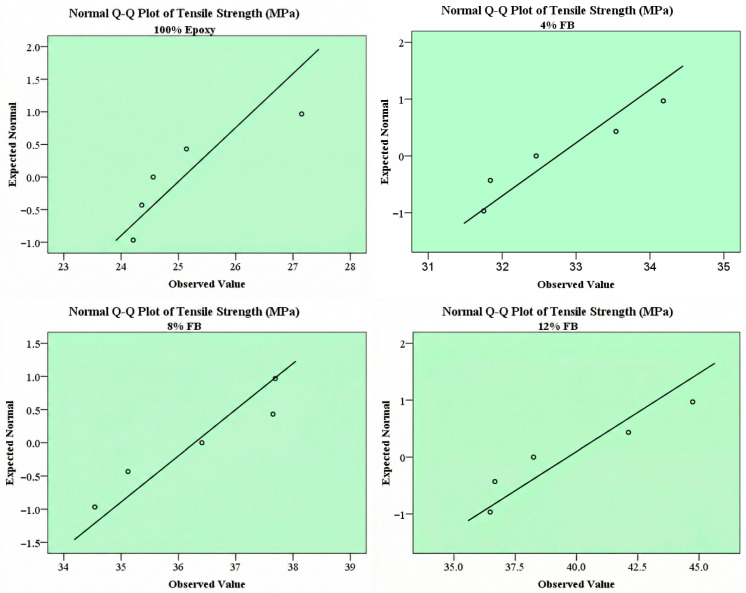
Normal Q–Q plots of tensile strength for each material group used to assess the normality assumption before one-way ANOVA.

Before inferential comparisons, the assumption of normality was assessed for each group using the Shapiro–Wilk test. The results showed no statistically significant deviation from normality in any of the groups (all *p* > 0.05); therefore, the assumption of normality was found to be acceptable for the current dataset (Table 2) [71]. Furthermore, considering the small sample size (n = 5), this interpretation was visually supported by group-based normality Q–Q plots (Figure 7).

The assumption of homogeneity of variance was then examined using the Levene test. The Levene test was found to be statistically significant (F(3,16) = 7.413, *p* = 0.002), indicating that the variances are not equal between the groups (Table 3). Therefore, although the classical one-way ANOVA result is significant, the main inferential interpretation is based on the Welch ANOVA result, which is more robust against variance heterogeneity [72,73]. The classical one-way ANOVA showed that the material group had a significant effect on tensile strength (F(3,16) = 43.579, *p* < 0.001). Consistently, the Welch ANOVA result also confirmed that the group effect was statistically significant (F(3,8.591) = 65.843, *p* < 0.001). These findings indicate that the FB ratio significantly affects tensile strength (Table 3). The effect size was found to be very high (η^2^ = 0.891; Table 3, Note), indicating that approximately 89.1% of the total variance in tensile strength is explained by the material group (FB ratio).

Since the assumption of homogeneity of variance was not met, the Games–Howell test was used instead of the Tukey HSD in post hoc comparisons [74,75]. Pairs-by comparisons revealed that all groups containing FB showed statistically significantly higher tensile strength compared to the 100% photopolymer group. Furthermore, the 8% FB and 12% FB groups also had significantly higher values compared to the 4% FB group. In contrast, the difference between the 8% FB and 12% FB groups was not statistically significant (*p* = 0.321); however, the mean value of the 12% FB group was higher (Table 4). This result suggests a strong improvement in tensile strength with the addition of FB, but that the performance increase in the 8–12% FB range may be approaching saturation or that there is overlap between groups due to increased variability.

From a materials engineering perspective, FB additive significantly improves tensile performance compared to pure Photopolymer. The observed increase and very large effect size when switching from the 100% Photopolymer group to the FB modified groups support the idea that FB reinforcement provides a mechanically significant contribution. However, the high variance observed in the 12% FB group suggests that at higher filler ratios, production homogeneity, filler distribution, interfacial bonding, or sample-based heterogeneity may become more decisive. Therefore, although 12% FB yielded the highest average tensile strength, considering the statistical overlap with 8% FB, not only average strength but also reproducibility and the level of variation should be evaluated together in the optimization process.

## 4. Conclusions

This study demonstrated the feasibility of using salmon bone ash, a biological waste-derived calcium-phosphate-based filler, as a sustainable reinforcement material for SLA-compatible photopolymer composites. Photopolymer composites containing 4, 8, and 12 wt.% fish bone ash were successfully produced after optimization of the SLA printing parameters, and their mechanical, chemical, and fracture characteristics were systematically evaluated.

The incorporation of fish bone ash significantly improved the tensile strength of the photopolymer matrix. The highest tensile strength was obtained at 12 wt.% filler loading, confirming the reinforcing effect of the rigid inorganic particles. However, increasing filler content reduced elongation at break and promoted a more brittle fracture response. Therefore, although 12 wt.% provided the maximum strength, the 8 wt.% composition exhibited the most balanced mechanical performance in terms of strength and deformation capacity. Statistical analysis using Welch ANOVA and Games–Howell post hoc testing confirmed that filler loading had a significant effect on tensile strength under heterogeneous variance conditions.

FTIR results showed that fish bone ash remained chemically stable within the photopolymer matrix and that the matrix–filler interactions were mainly physical rather than covalent. SEM observations supported the mechanical findings by showing that low and medium filler loadings promoted rougher fracture surfaces, crack deflection, and energy dissipation, whereas higher filler loading increased particle agglomeration, microvoid formation, and brittle fracture tendency. These findings indicate that the composite performance depends not only on filler content but also on particle dispersion and matrix–filler interfacial compatibility.

Overall, salmon bone ash shows considerable potential as an environmentally sustainable and mechanically effective bio-waste-derived filler for SLA photopolymer composites. This study contributes to the valorization of fish-processing waste in additive manufacturing applications. Future studies should focus on particle size control, surface modification, resin rheology, thermal and dynamic mechanical behavior, and comparative evaluation of fish bone ash obtained from different fish species using quantitative characterization techniques such as XRF, EDS, XRD, and particle size analysis.

## Figures and Tables

**Table 1:** Tensile strength and elongation values of neat photopolymer and photopolymer/FB ash composite samples.

Material	Stress (MPa)	Strain (%)
Photopolymer	24.8 ± 1.2	8.37 ± 0.4
Photopolymer + 4 wt.% FB ash	32.4 ± 1.6	4.02 ± 1.1
Photopolymer + 8 wt.% FB ash	35.7 ± 1.8	5.16 ± 0.3
Photopolymer + 12 wt.% FB ash	37.9 ± 1.9	4.21 ± 0.2

**Table 2:** Descriptive statistics and normality test results for tensile strength by material groups.

Material	n	Mean (MPa)	SD	Shapiro–Wilk W	*p*-Value
100% Photopolymer	5	25.084	1.208	0.787	0.063
4% FB	5	32.754	1.070	0.895	0.385
8% FB	5	36.282	1.437	0.886	0.337
12% FB	5	37.952	3.637	0.876	0.289

**Table 3:** Homogeneity and ANOVA/Welch ANOVA results for tensile strength.

Test	Statistic	df1	df2	*p*-Value	Interpretation
Levene’s test	7.413	3	16	0.002	Variances not homogeneous
One-way ANOVA	43.579	3	16	<0.001	Significant group effect
Welch ANOVA	65.843	3	8.591	<0.001	Significant group effect (robust)

Note: Effect size for the material group was large (η^2^ = 0.891), calculated from the classical one-way ANOVA sums of squares as $\eta^{2}=SS_{\mathrm{between}}/SS_{\mathrm{total}}$. Although Levene’s test indicated heterogeneity of variances, Welch ANOVA was used for the primary inferential decision, while η^2^ is reported to quantify the magnitude of the group effect.

**Table 4:** Games–Howell post hoc comparisons for tensile strength.

Comparison	Mean Difference (MPa)	*p*-Value
100% Photopolymer—4% FB	−7.670	<0.001
100% Photopolymer—8% FB	−11.198	<0.001
100% Photopolymer—12% FB	−14.568	0.002
4% FB—8% FB	−3.528	0.012
4% FB—12% FB	−6.898	0.039
8% FB—12% FB	−3.370	0.321

## Data Availability

The original contributions presented in the study are included in the article. Further inquiries can be directed to the corresponding author.
